# Comparative transcriptome analysis identifies *CARM1* and *DNMT3A* as genes associated with osteoporosis

**DOI:** 10.1038/s41598-020-72870-2

**Published:** 2020-10-01

**Authors:** Layla Panach, Clara Pertusa, Beatriz Martínez-Rojas, Álvaro Acebrón, Damián Mifsut, Juan J. Tarín, Antonio Cano, Miguel Ángel García-Pérez

**Affiliations:** 1 Research Unit, INCLIVA Health Research Institute, 46010 Valencia, Spain; 2 Orthopedic Surgery and Traumatology, Clinic Hospital, Institute of Health Research INCLIVA, 46010 Valencia, Spain; 3 Department of Cellular Biology, Functional Biology and Physical Anthropology, University of Valencia, 46100 Burjassot, Spain; 4 Department of Pediatrics, Obstetrics and Gynecology, University of Valencia, 46010 Valencia, Spain; 5 Department of Genetics, University of Valencia, 46100 Burjassot, Spain

**Keywords:** Gene expression, Genetic association study, Genotype, Quantitative trait, Endocrine system and metabolic diseases

## Abstract

To identify new candidate genes in osteoporosis, mainly involved in epigenetic mechanisms, we compared whole gene-expression in osteoblasts (OBs) obtained from women undergoing hip replacement surgery due to fragility fracture and severe osteoarthritis. Then, we analyzed the association of several SNPs with BMD in 1028 women. Microarray analysis yielded 2542 differentially expressed transcripts belonging to 1798 annotated genes, of which 45.6% (819) were overexpressed, and 54.4% (979) underexpressed (fold-change between − 7.45 and 4.0). Among the most represented pathways indicated by transcriptome analysis were chondrocyte development, positive regulation of bone mineralization, BMP signaling pathway, skeletal system development and Wnt signaling pathway. In the translational stage we genotyped 4 SNPs in *DOT1L*, *HEY2*, *CARM1* and *DNMT3A* genes. Raw data analyzed against inheritance patterns showed a statistically significant association between a SNP of *DNMT3A* and femoral neck-(FN) sBMD and primarily a SNP of *CARM1* was correlated with both FN and lumbar spine-(LS) sBMD. Most of these associations remained statistically significant after adjusting for confounders. In analysis with anthropometric and clinical variables, the SNP of *CARM1* unexpectedly revealed a close association with BMI (*p* = 0.000082), insulin (*p* = 0.000085), and HOMA-_IR_ (*p* = 0.000078). In conclusion, SNPs of the *DNMT3A* and *CARM1* genes are associated with BMD, in the latter case probably owing to a strong correlation with obesity and fasting insulin levels.

## Introduction

Osteoporosis is a common skeletal age-related disease characterized by low bone mass and deterioration in bone micro-architecture, leading to compromised bone strength and increased risk of fragility fractures^1^. As with other degenerative diseases, its increasing prevalence as a result of greater life expectancy has dramatic socioeconomic repercussions due to the high morbi-mortality of its direct clinical consequence, bone fracture caused by fragility^2^.

Although they are prevalently female pathologies, osteoporosis and bone fracture due to fragility affect both sexes. Aside from the estrogenic depletion that occurs in women during menopause, the etiology of excessive bone loss that leads to osteoporosis includes several age-related factors common to both sexes, such as hyperparathyroidism, vitamin D resistance and decreased antioxidant capacity^3^.

Osteoporosis and fragility fracture are complex phenotypes of multifactorial origin, governed by both genetic and environmental influences as well as their interactions. The main clinical risk factors are well known, particularly insufficient calcium and vitamin D, low or no physical activity, low BMD and intake of toxic substances such as alcohol or tobacco. Many of these factors appear among the main parameters of fracture risk estimation algorithms such as the FRAX and Garvan tools^4^.

Like other multifactorial pathologies, osteoporosis and fragility fracture are caused by multiple common variants, each having a small contribution to total phenotypic variance^5^. However, after several years conducting allelic association studies either with (candidate gene approach) or without (GWAS approach) a previous hypothesis, currently known susceptibility loci can still only explain about 20% of estimated heritability for BMD^6^ and 2% for fragility fracture^7^, a problem known as missing heritability^8^. Given that increasing the sample size has limited effectiveness in identifying missing heritability^9^, other approaches have also been developed, such as study of rare, structural or epigenetic variants^5,8^. However, although various epigenetic mechanisms such as microRNAs, long non-coding RNA, gene expression and DNA methylation are involved, the exact percentage in which bone phenotypes variances can be attributed to epigenetic variants is unknown^7^. Similarly, several rare variant association studies have detected variants with important effects on bone phenotypes, although these only explain a tiny proportion of the phenotypic variance since the heritability explained is dependent on effect size and allele frequency^10^.

We have previously used translational approaches from animal models to identify potential candidate genes that help to explain this missing heritability^11,12^. The rationale behind this methodological approach was to compare whole gene expression in the animal model in situations of bone loss with that of control animals to identify genes with differential expression between groups. Later, we translated the study to humans, analyzing the association of several SNPs in any of these differentially expressed genes (DEG) with bone phenotypes, which led us to identify certain genes with bone phenotypes, such as *CD80* and *CD79A*^11,12^.

In the present study we also carried out a translational approach from a human cellular model. To this end, we used microarrays to analyze and compare whole gene expression in primary osteoblasts obtained from femoral heads of women undergoing hip replacement surgery due to a fragility fracture or severe osteoarthritis. Next we examined the association between BMD and SNPs in four candidate genes. In this study we focused on epigenetic-related genes for candidate genes selection, for two main reasons: (i) increasing evidence that several epigenetic mechanisms are involved in bone metabolism, leading to the conviction that osteoporosis has a strong epigenetic component^13^, and (ii) epigenetic variant research is a well-established approach in the search for missing heritability^14^.

## Materials and methods

### Subjects

Primary osteoblasts (OBs) were obtained from women of Caucasian ethnicity undergoing a hip replacement at the Hospital Clínico Universitario de Valencia, due either to subcapital hip osteoporotic fracture (Fracture group, N = 6) or severe hip osteoarthritis (Control group, N = 6). Women with other bone pathologies or fractures due to high-energy trauma or cancer were excluded.

For BMD association studies, we analyzed a cohort of Caucasian women living in Valencia previously enrolled in studies related to women's health^11,12,15^. These women were recruited consecutively from the Menopause Unit of the Hospital Clínico and the Hospital Doctor Peset, due to climacteric-related problems, which was the only inclusion criterion for the study. A total of 1275 women gave informed consent to be included in the study.

Exclusion criteria in this study were (i) history of bone disease other than primary osteoporosis, (ii) hyperparathyroidism, hyperthyroidism, renal insufficiency, primary amenorrhea or serious neurological illness such as Parkinson or Alzheimer disease (iii) chemotherapy before densitometric study (iv) previous corticosteroid treatment, and (v) under 35 years of age. Bone active treatment, such as hormone therapy (HT) or bisphosphonates, was considered as a covariate, but calcium and vitamin D supplementation was not included as a bone active treatment. After applying exclusion criteria, 1028 women were finally included in the study.

Relevant patient data such as age, weight, height, waist and hip circumference, use of bone active treatment, etc. was collected as previously described^12^. The Medicaments Research Ethics Committee (CEIm) of the Hospital Clínico Universitario of Valencia approved the study in accordance with the principles of the Declaration of Helsinki, and written informed consent was obtained from subjects in accordance with INCLIVA Health Research Institute guidelines.

### Characterization of primary osteoblasts

The femoral head was obtained during surgery and placed in a sterile bottle at 4ºC until processed once surgery was completed. Bone cylinders 1 cm in diameter were obtained from the central part of the femoral head with a trephine, avoiding the fracture zone. Once the cortical area was removed, cylinders were washed thoroughly with PBS supplemented with 1% Fungizone (Gibco) to remove bone marrow remains. The cylinders were then cut into discs about 2–3 mm thick and 3–4 bone fragments were seeded into T-25 plastic flasks containing Dulbecco's Modified Eagle's Medium, 20% FBS, 1% antibiotics and 1% Fungizone. Under these conditions OBs precursor cells can migrate from bone fragments and proliferate^16^. After confluence, cells were trypsinized to subculture or to obtain RNA.

To verify the osteoblastic origin of the cultures, osteocalcin expression was analyzed using flow cytometry. Cells were collected with Accutase (BD, San José, CA, USA), then fixed by Cytofix (Fixation Buffer, BD) and permeabilized using Phosflow Perm Buffer III (BD). Next the samples were labeled with phycoerythrin Mouse Anti-Human Osteocalcin (BD). At the time of obtaining RNA from cultures, about 85% of the cells were positive for this marker (not shown).

### RNA isolation and GeneChip expression analysis

Total RNA was extracted from OBs using the TRIzol reagent (Invitrogen) following the manufacturer’s instructions. RNA integrity number (RIN) was determined by 2100 Bioanalyzer (Agilent Technologies, Santa Clara, CA, USA), and RNA concentration was determined using a highly sensitive capillary spectrophotometer (GeneQuant, GE Healthcare Biosciences). For transcriptome analysis we used the GeneChip™ Human Gene 2.0 ST Array (Affymetrix), with 6 arrays per group, which includes 1,350,000 probes for 40,716 RefSeq transcripts and 11,086 lincRNA transcripts.

cDNA and cRNA synthesis, labelling, hybridization and scanning of the samples was performed in accordance with the WT Plus Reagent Kit Manual (Affymetrix Ltd, UK), as previously described^11,12^. After scanning, the generated CEL-files were examined using Transcriptome Analysis Console (TAC) software (version 4.0.1) (Applied Biosystems) to analyze gene expression and visualize the results. The genes were filtered by fold-change value (± 1.0). All other parameters selected were default parameters in the TAC software. The list of DEG was processed by the GeneCodis3 web tool (https://genecodis.genyo.es) to analyse altered biological processes^17^, and the PANTHER classification system was used for large-scale genome and gene function analysis^18^.

The raw microarray data files were submitted to the Gene Expression Omnibus (GEO) repository (accession number GSE 156508).

### Microarray data validation by MassARRAY-QGE and real-time qPCR

Results from microarray experiments were validated using MALDI-TOF mass spectrometry technology (Sequenom, CA, USA). We analyzed 4 replicates of 12 RNAs for 12 genes using 8 competitor dilutions in each experiment, and 4 housekeeping genes (*GAPDH*, *HPTR1*, *ACTB*, and *HMB2*) were also used.

PCR primers and competitors for each gene (Supplemental Table S1) were designed using Sequenom software. One microgram of total RNA was reverse transcribed using the High Capacity RNA-to-cDNA Kit (Thermo Fisher Scientific, Waltham, MA, USA) with a combination of random octamers and oligo(dT). Competitive PCR, removal of excess dNTPs, primer extension reaction and RNA quantification were performed as described^11^.

### Biochemical assays

Carboxy-terminal telopeptides levels of collagen I (CTx) and insulin were measured by electrochemiluminescence (E170 Modular Analyser; Roche Diagnostics, Mannheim, Germany). Levels of total alkaline phosphatase (ALP), total calcium and phosphate, glucose, triglycerides, total cholesterol and high-density lipoprotein cholesterol (HDL) were assessed using a spectrophotometer (Olympus 5400, Olympus, Melville, NY, USA). Low-density lipoprotein cholesterol (LDL-cholesterol, mg/dL) was calculated as TC − (HDL + TG/5)^19^. The HOMA-_IR_ (homeostasis model assessment) insulin resistance index was calculated as fasting serum insulin in µIU/mL x (fasting serum glucose in mg/dL × 0.05551)/22.5^20^.

### Bone mineral density (BMD) data

A densitometric study was performed at femoral neck (FN-BMD) and/or lumbar spine L2-L4 (LS-BMD) sites by dual energy X-ray absorptiometry (DXA). In the present study we used a Norland XR-36 (Norland Medical Systems Inc; Fort Atkinson, WI, USA), Lunar DPX (GE Lunar Corporation, Madison, WI, USA), or Hologic (Hologic Explorer TM Explorer Series, Marlborough, MA, USA) densitometer, and a standardized BMD (sBMD) was calculated^21,22^.

### Single nucleotide polymorphisms (SNPs) and genotyping

In the present study we analysed the association of SNPs in four genes with bone phenotypes (Table 1). Three of the four genes analyzed (*DOT1L*, *HEY2*, and *CARM1*) were selected from the list of DEG in OBs of subjects with fracture compared with those in the control group, after filtering for genes with epigenetic involvement (Supplemental Table 2). From the epigenetic-related gene list we conducted an exhaustive bibliographic search to find the three with most accumulated evidence of involvement in bone processes. The four candidate genes chosen were: *DOT1L*, *HEY2*, *CARM1* and *DNMT3A*. *DOT1L* gene (DOT1 Like Histone Lysine Methyltransferase; downregulated in the Fracture group) encodes for a histone methyltransferase that inhibits osteoclastogenesis^23^, and prevents hyperactivation of Wnt signalling^24^, a main pathway involved in OB differentiation. *HEY2* gene (Hes Related Family BHLH Transcription Factor With YRPW Motif 2; upregulated in the Fracture group) encodes for a downstream effector of Notch signalling and is implicated in OB differentiation by controlling the stability of Hes1-Runx2 complexes and thus affecting expression of their target genes^25^. *CARM1* gene (Coactivator Associated Arginine Methyltransferase 1; downregulated in the Fracture group) encodes for a histone methyltransferase that regulates the enzyme 25-Hydroxyvitamin D_3_ 24-Hydroxylase which is essential in bone catabolism^26^. Finally, *DNMT3A* gene (DNA Methyltransferase 3 Alpha) which encodes for a de novo DNA methyltransferase, was chosen as a candidate gene for its regulatory role during osteoclast differentiation^27^, and for being one of the targets of miRNA-143, a miRNA previously associated with bone fracture by our group^28^. As far as we know, to date none of these genes have previously been studied for association with BMD in a human cohort.

**Table 1:** Single nucleotide polymorphisms (SNPs) studied in the cohort. *p* values were obtained from Chi-square test.

Chromosome	SNP	Gene	Position (GRCh38.p12)	Location	Major allele	Minor allele	MAF	*p* HWE
19	rs11880992	*DOT1L*	2,176,404	Intronic	G	A	0.46	0.21
6	rs9388451	*HEY2*	125,769,231	Intergenic	C	T	0.49	0.029
19	rs12460421	*CARM1*	10,870,676	2 KB Upstream Variant	A	G	0.45	0.002
2	rs6722613	*DNMT3A*	25,316,488	Intronic	A	G	0.41	0.2

**Table 2:** Anthropometric and bone characteristics of the cohort studied (mean ± SD or percentage). N = 1028, except for percentage of postmenopausal women (N = 991) and percentage of antiresorptive therapy user (N = 1009).

	**Values**
Age (y)	55.1 ± 8.6
Weight (kg)	66.3 ± 10.4
Height (cm)	157.6 ± 6.0
BMI (kg/m^2^)	26.8 ± 4.2
Waist circumference (cm)	85.7 ± 10.4
Hip circumference (cm)	102.3 ± 8.5
Postmenopausal women (%)	93.2
Antiresorptive therapy user (%)	24.7
FN-BMD (g/cm^2^)	0.799 ± 0.118
FN T-score	− 0.978 ± 1.011
FN Z-score	− 0.070 ± 0.944
LS-BMD (g/cm^2^)	0.993 ± 0.150
LS T-score	− 1.169 ± 1.351
LS Z-score	− 0.139 ± 1.224

*BMI* body mass index, *HT* hormone therapy, *BMD* bone mineral density, *FN* femoral neck, *LS* lumbar spine.

The SNPs of the study were chosen based on adequate heterozygosity and a minor allele frequency (MAF) of > 5% in Iberian populations in Spain (IBS). Furthermore, we used the Ensembl genome browser to detect regulatory variants or SNPs previously associated with other phenotypes. The selected SNPs were: SNP rs11880992 (*DOT1L* gene; associated with bone phenotype and height), SNP rs9388451 (*HEY2* gene; regulatory region variant associated with Brugada syndrome), SNP rs12460421 (*CARM1* gene; regulatory region variant associated with LDL levels), and SNP rs6722613 (*DNMT3A* gene; associated with ossification of the posterior longitudinal ligament of the spine).

DNA extraction from blood samples, genotyping of the SNPs by allelic discrimination, and quality control was performed as previously described^12^.

### Statistical analysis

Comparative transcriptomic analysis of altered gene expression processes and pathways between the two groups was performed using TAC software tools. For normalization we selected the Robust Multi-Chip Analysis (RMA) algorithm which performs background adjustment, quantile normalization and summarization. All samples passed the quality control to detect possible outliers performed using PCA analysis and hierarchical clustering. A one-way ANOVA was used to detect differentially expressed genes (DEG) with False Discovery Rate (FDR) as multiple testing correction. A *p* value < 0.05 was considered statistically significant.

The Student t test was used to validate statistically significant genes in the microarray experiment using MALDI-TOF mass spectrometry.

With regard to genotyping, we used the SNPStats software (https://www.snpstats.net/start.htm) to test the homogeneity of the population by determining the genotypic frequencies for each SNP against the Hardy–Weinberg Equilibrium (HWE) and to estimate the best inheritance model (co-dominant, dominant, overdominant or recessive)^29^.

In order to minimize the influence of outliers detected by Tukey’s test, quantitative measured variables (Table 5) were winsorized (i.e. the data point was replaced with the next highest or lowest non-outlier value). We assumed missing at random, but to adjust for potential bias associated with quantitative missing data, 5 imputations were considered and pooled estimates of these imputations were used for statistical analyses. Fixed-effects analysis of variance (ANOVA) designs were used to compare means between genotype groups. Analysis of covariance (ANCOVA) was used to examine differences in the dependent variable (sBMD) among genotypes after adjustment for confounding variables. Age and BMI were considered as covariates. Given their dichotomous nature, postmenopausal status (yes/no) and antiresorptive therapy user (yes/no) were entered into statistical models as fixed factors.

Multiple linear regression model with stepwise variable selection were performed to detect linear relationships with the dependent variable (insulin level), with adjustment for independent variables (age, weight, BMI, waist and hip circumferences, waist to hip ratio, SNP, lipids, and glucose). The SNP rs12460421 of *CARM1* gene was introduced as a dummy variable and codified as 0 or 1 (0 genotype GA/AA, and 1 genotype GG, according to inheritance model). To avoid multicollinearity, we removed highly correlated predictors from the model, excluding all independent variables whose variance inflation factor (VIF) was > 5 (weight, waist circumference and total cholesterol). All independent variables in the final model in Table 6 showed VIF values < 2, indicating low/moderate collinearity^30^.

We estimated a sample size of 965 would be sufficient to achieve 80% statistical power using the QUANTO software package (https://hydra.usc.edu/GxE/). This was calculated considering a gene effect of R^2^ = 0.008 (obtained from regression analysis between LS-BMD and rs12460421), according to a G allele frequency of 0.45 (Table 1) under a recessive model of inheritance and a mean ± SD for LS-BMD of 0.993 ± 0.151 (Table 2).

We used heatmap to visualize correlation (Supplemental Figure S1), using the Pearson correlation coefficient matrix calculated in R (Version 3.6.2, GNU project, The R Project for Statistical Computing).

All analyses were two-tailed, and statistical significance was defined as *p* < 0.05, except for multiple comparisons, in which the cut-off value for the Bonferroni correction was estimated as *p* < 0.0063 (0.05/8; two phenotypes, LS-sBMD and FN-sBMD, and four SNPs). Data was analysed using IBM SPSS statistics for Windows (v.24.0; Armonk, NY: IBM Corp.).

### Results

### Differentially expressed genes between fracture and control groups of women

OBs were obtained from women of a similar age (Fracture: 68.8 ± 7.3 years vs. control: 73.5 ± 9.8 years, *p* = 0.37), but differing hip BMD (Fracture FN Z-score: − 0.367 ± 0.616 vs control FN Z-score: 2.125 ± 1.190, *p* = 0.002).

**Figure 1:**
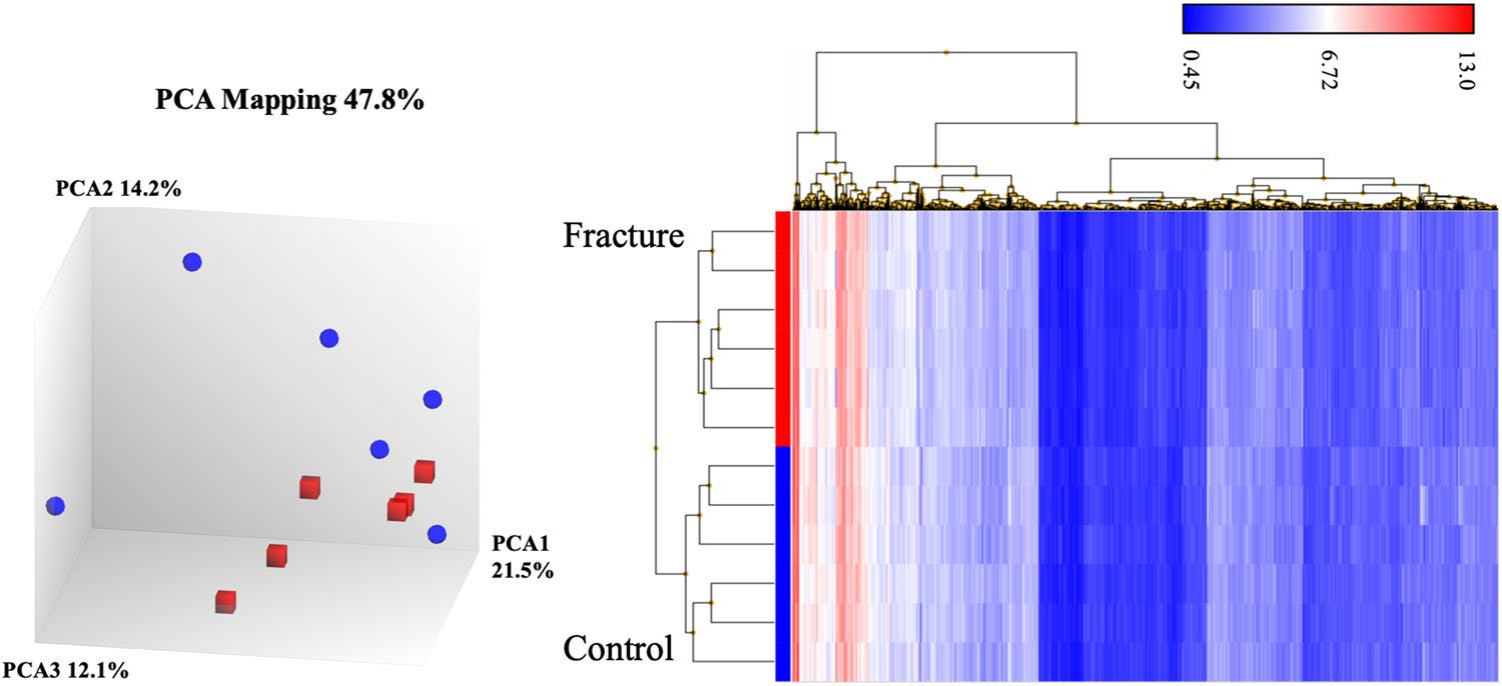
(**A**) Principal components analysis (PCA) and (**B**) hierarchical clustering of transcriptome data of OBs from Fracture (blue, N = 6) and Control (red, N = 6) women.

Figure 1 shows principal component analysis (PCA) and hierarchical clustering of transcriptome in expression microarray data of OBs obtained from Fracture and Control groups of women. PCA captures the variance of the principal components, and in our study (Fig. 1A) shows that overall gene expression is different across the two groups, although OB data from women with fracture are more homogeneous. Despite this greater heterogeneity in the control group, since they all passed the TAC software quality controls we decided to use them all in subsequent analysis. Our observation that gene expression is substantially different across the two groups can be better appreciated from hierarchical clustering analysis (Fig. 1B), which shows that RNA samples from OBs of women with fracture are separated in a different cluster to those of the control women, indicating that there are two clearly different groups regarding gene expression.

To perform DEG analysis, data from the 12 microarrays were analyzed with TAC software, showing 2542 statistically significant transcripts belonging to 1798 annotated genes (Supplemental Table S2), of which 819 were upregulated (45.6%) and 979 (54.4%) downregulated. Given that we used OBs from bone samples, these differentially expressed transcriptomic signatures should primarily refer to osteoporosis, as inflammatory osteoarthritis is principally a joint disease.

#### DEG identification and functional classification

The altered Gene Ontology-based biological process pathways were analysed with the GeneCodis3 web tool (Supplemental Table S3), yielding 182 GO terms that passed the False Discovery Rate (FDR) test. These include chondrocyte development (6 annotated genes in the input list/17 annotated genes in the reference list; *p* = 0.000015); positive regulation of bone mineralization (8/35, *p* = 0.00002); BMP signaling pathway (11/79, *p* = 0.00008); skeletal system development (15/145, *p* = 0.00015); positive regulation of chondrocyte differentiation (5/20, *p* = 0.00048); intramembranous ossification (3/6, *p* = 0.0008); ossification (9/73, *p* = 0.0009); negative regulation of BMP signaling pathway (7/46, *p* = 0.0009); endochondral ossification (5/25, *p* = 0.0014); positive regulation of canonical Wnt signaling pathway (10/93, *p* = 0.0014); cartilage development (8/63, *p* = 0.0014), and bone development (6/39; *p* = 0.002).

The DEG list was analyzed with the PANTHER (protein analysis through evolutionary relationships) classification system^18^ to classify and identify the function of gene products in Fracture OBs (Supplemental Table S4). The categories with the highest percentage of genes present in the DEG list were inflammation mediated by chemokine and cytokine signaling pathway (26 genes, 5.9% percent of gene hit against total pathway hits), integrin signaling pathway (18 genes, 4.1%), Wnt signaling pathway (17 genes, 3.8%), FGF signaling pathway (10 genes, 2.3%) and TGF-beta signaling pathway (9 genes, 2%).

### Microarray data validation by MassARRAY QGE

To validate the results obtained from microarray analysis, we used MassARRAY-QGE analysis to determine the expression of 12 genes that were statistically significant in the microarray study, using the same RNAs as in the microarray experiment (Table 3). As can be seen, the fold change of the Sequenom study was similar to the fold change obtained with TAC software. A total of 9 genes (75%) showed statistically significant differences between groups (Spearman’s Rho = 0.87, *p* = 0.0003), demonstrating that the microarray study results represent a true approximation of gene expression status in each OB group^31^.

**Table 3:** Validation of microarray results by MassARRAY QGE analysis (Sequenom), showing the fold change obtained from microarray analysis with TAC software and with Sequenom analysis. The fold change from Sequenom is obtained by dividing the molar concentration of each gene between Fracture and Control groups of women (six women each). *p* value for Sequenom analysis was obtained using a paired Student’s t test.

Gene	Fold change from microarrays (TAC)	Fold change from Sequenom	*p* value
*SULF2*	2.53	2.09	0.010
*DSG3*	1.83	1.99	0.013
*COL10A1*	1.78	2.35	0.005
*RSPO3*	1.69	2.03	0.024
*MEF2C*	1.55	1.28	0.507
*ADAM12*	− 1.68	− 1.86	0.044
*GALNT5*	− 1.77	− 1.13	0.682
*THSD4*	− 1.78	− 1.77	0.005
*ITGA4*	− 2.07	− 1.68	0.008
*FOSL1*	− 2.31	− 1.49	0.418
*IL13RA2*	− 2.47	− 2.84	0.002
*LEP*	− 2.61	− 2.84	0.00001

### Association of SNPs with BMD

To translate these findings to our cohort of women, we selected four SNPs from the DEG list (main characteristics in Table 1) in four genes related to epigenetic mechanisms.

The SNP genotyping call rate was higher than 95%. As shown in Table 1, MAF was over 5% for all SNPs, and two of them deviated from the HWE (rs9388451 in *HEY2* gene, *p* = 0.029; and rs12460421 in *CARM1* gene, *p* = 0.002).

Analysis after applying multiple imputation did not substantially modify the results obtained in previous analyses using complete cases (data not shown). Participating subjects had a mean age of 55.1 years, with Z-score values indicating normal bone status for age, and BMI in the overweight range (Table 2).

Table 4 shows raw and estimated sBMD values adjusted for age, BMI, postmenopausal status and therapy-user covariates, by genotype according to inheritance pattern. With the raw data, the SNP rs6722613 of the *DNMT3A* gene was marginally associated with FN-sBMD (*p* = 0.021, η^2^ = 0.005), while the SNP rs12460421 of the *CARM1* gene was more strongly associated with both FN-sBMD (*p* = 0.0068, η^2^ = 0.007) and LS-sBMD (*p* = 0.0057, η^2^ = 0.007). When adjusted for confounding variables, both associations, SNP of *DNMT3A* gene with FN-sBMD and SNP of *CARM1* gene with LS-sBMD, were retained (Table 4). In addition, in a logistic regression study in which the dependent variable was hip osteoporosis + osteopenia vs. normal and adjusted for the same covariates, we obtained an OR of 1.46 for the A/G–G/G genotype of SNP of *DNMT3A* gene (95% CI: 1.110–1.929, *p* = 0.007, not shown).

**Table 4:** Analysis of the association between genotypes and sBMD, unadjusted and adjusted for confounding variables: age, BMI, postmenopausal status and treatment use. Unadjusted values are means ± SD. Adjusted values are estimated means ± SE. Unadjusted and adjusted *p* values were obtained con ANOVA and ANCOVA tests, respectively.

Gene SNP (rs)	Genotype	FN-sBMD		Genotype	LS-sBMD	
		Unadjusted (N)	Adjusted (N)		Unadjusted (N)	Adjusted (N)
***HEY2*** rs9388451	C/C	0.790 ± 0.122 (247)	0.812 ± 0.011 (247)	C/C	0.983 ± 0.157 (247)	1.023 ± 0.014 (247)
	C/T–T/T	0.802 ± 0.116 (777)	0.815 ± 0.010 (777)	C/T–T/T	0.996 ± 0.148 (777)	1.030 ± 0.013 (777)
	*p* value	0.166	0.711	*p* value	0.234	0.531
***DNMT3A*** rs6722613	A/A	0.811 ± 0.120 (350)	0.827 ± 0.011 (350)	A/A–A/G	0.995 ± 0.151 (861)	1.030 ± 0.012 (861)
	A/G–G/G	0.793 ± 0.116 (670)	0.809 ± 0.010 (670)	G/G	0.983 ± 0.143 (159)	1.015 ± 0.017 (159)
	*p* value	0.021	0.018	*p* value	0.358	0.241
***DOT1L*** rs11880992	A/G–G/G	0.801 ± 0.117 (816)	0.817 ± 0.010 (816)	A/G–G/G	0.991 ± 0.148 (816)	1.028 ± 0.012 (816)
	A/A	0.791 ± 0.117 (206)	0.804 ± 0.012 (206)	A/A	1.001 ± 0.155 (206)	1.030 ± 0.015 (206)
	*p* value	0.255	0.118	*p* value	0.386	0.817
***CARM1*** rs12460421	A/A–A/G	0.794 ± 0.117 (785)	0.810 ± 0.010 (785)	A/A–A/G	0.986 ± 0.147 (785)	1.022 ± 0.013 (785)
	G/G	0.817 ± 0.119 (233)	0.826 ± 0.011 (233)	G/G	1.017 ± 0.156 (233)	1.045 ± 0.015 (233)
	*p* value	0.0068	0.060	*p* value	0.0057	0.040

In light of these results, we then analyzed anthropometric and biochemical variables as regarded the genotypes of the *DNMT3A* and *CARM1* genes. Supplemental Figure S1 shows a correlation matrix (Pearson coefficient) obtained with the values of the variables in Table 5 together with FN- and LS-BMD. No noteworthy findings were obtained for the *DNMT3A* gene, but we found an association between the *CARM1* SNP genotype and anthropometric and biochemical variables (Table 5). Indeed, homozygous women for the G allele showed considerably higher weight, BMI, waist and hip circumferences, insulin levels and HOMA-_IR_ index than the GA/AA women. Also, the women of the GG genotype showed higher levels of calcium, triglycerides and glucose levels than women of the GA/AA genotype. Most of these associations remained statistically significant even after applying the Bonferroni coefficient, assigning a threshold value of *p* < 0.0028 (0.05/18 outcomes) for the variables in Table 5. Consequently, there were no differences in height, glucose levels, or body fat distribution as measured by waist-to-hip ratio.

**Table 5:** Anthropometric and biochemical data of the study cohort by inheritance pattern for SNP rs12460421 of *CARM1* gene. Values are mean ± SD. *p* value was obtained with an ANOVA test. N = 1018.

	Genotypes (N)		*p* value
	GG (233)	GA/AA (785)	
Age (y)	54.8 ± 8.2	55.1 ± 8.7	0.598
Weight (kg)	68.7 ± 10.7	65.6 ± 10.2	0.000082
Height (cm)	157.9 ± 6.4	157.4 ± 6.0	0.301
BMI (kg/m^2^)	27.7 ± 4.5	26.5 ± 4.1	0.00022
Waist circumference (cm)	87.5 ± 11.1	85.2 ± 10.2	0.0023
Hip circumference (cm)	104.0 ± 8.9	101.8 ± 8.4	0.00085
Waist to hip ratio	0.841 ± 0.067	0.836 ± 0.067	0.307
CTx (ng/mL)	0.428 ± 0.178	0.441 ± 0.191	0.346
Total ALP (U/L)	168.2 ± 45.0	168.0 ± 45.5	0.942
Calcium (mg/dL)	9.6 ± 0.4	9.6 ± 0.4	0.011
Phosphate (mg/dL)	3.6 ± 0.5	3.6 ± 0.5	0.938
Total cholesterol (mg/dL)	217.3 ± 32.2	215.4 ± 34.3	0.444
HDL-cholesterol (mg/dL)	63.9 ± 12.9	64.2 ± 14.0	0.788
LDL-cholesterol (mg/dL)	132.2 ± 27.6	131.5 ± 28.8	0.737
Triglycerides (mg/dL)	106.4 ± 39.2	99.4 ± 38.4	0.015
Glucose (mg/dL)	101.8 ± 11.5	100.0 ± 11.4	0.033
Insulin (μU/ml)	9.1 ± 4.1	8.0 ± 3.5	0.000085
HOMA-_IR_ index	2.3 ± 1.1	2.0 ± 1.0	0.000078

*CTx* carboxyterminal telopeptides of collagen I; *ALP* alkaline phosphatase; *HOMA-*_*IR*_ insulin resistance index (homeostasis model assessment).

Given that the *CARM1* gene has been associated with insulin secretion^32^, we conducted a multivariate regression study to detect if the *CARM1* SNP was independently associated with insulin level if confounder variables such as anthropometric data, lipids or fasting glucose, were included in the model. As seen in Table 6 the *CARM1* SNP association remained statistically significant after adjustment.

**Table 6:** Insulin level predictors determined by stepwise multiple linear regression analysis (N = 1018, in the final model).

Dependent variable	Independent variables	Unstandardized coefficients		*t*	*p* value	Adjusted R^2^
		B	SE			
Insulin	Intercept	− 5.024	1.198	− 4.194	< 0.0000	0.318
	BMI	0.252	0.025	10.176	< 0.0000	
	Triglycerides	0.026	0.003	9.367	< 0.0000	
	Glucose	0.049	0.009	5.551	< 0.0000	
	HDL	− 0.018	0.008	− 2.355	0.019	
	*CARM1* SNP	0.505	0.229	2.208	0.027	

## Discussion

In the present study, we describe the association between SNP rs6722613 of the *DNMT3A* gene and FN-sBMD, and between SNP rs12460421 of the *CARM1* gene and LS-sBMD. However, our main finding has been the discovery of a strong association between a SNP of the *CARM1* gene and BMI and fasting insulin levels, in addition to other related factors. Indeed, as regards the SNP G allele of the *CARM1* gene, homozygous women showed considerably higher BMI (*p* = 0.00022), insulin (*p* = 0.000085), waist circumference (*p* = 0.0023) and HOMA-_IR_ index (*p* = 0.000078) than GA/AA genotype women. This is the first time that normal genetic variation in these genes has been related to bone mass, and moreover, of great potential interest in cardiovascular risk assessment, the first time that genetic variants in *CARM1* gene have been correlated with obesity and fasting insulin levels. Despite conducting an exhaustive search in the GWAS catalog (www.ebi.ac.uk/gwas/) in a region of 500 Kb around the *CARM1* gene, we found no SNPs associated with BMI or fasting insulin levels.

As the main aim this study sought to translate results from a cellular to human model to identify new candidate genes for osteoporosis in women, as previously done by our group from animal models^11,12^. Here, we compared gene expression in OBs obtained from women with osteoporotic hip fracture and from control women with severe osteoarthritis. Although use of human OBs is a strength of this study, it might not be the optimal model for a posteriori research into these genes’ association with BMD, because although OB donors in the Fracture group had lower BMD (*p* = 0.002), the differentiating factor is fracture and not BMD. However, it is well known that BMD is the primary predictor of bone fracture^33^ and in transcriptome analysis in the two OB types, pathways involved in bone metabolism and BMD acquisition were consistently among the most altered and/or represented pathways, among which were positive regulation of bone mineralization, BMP signaling pathway, skeletal system development, intramembranous and endochondral ossification and Wnt signaling pathways.

Two of the genes analyzed in this study appear to be clear candidates for study of bone phenotypes. One of them, *DNMT3A* gene, which encodes for a de novo DNA methyltransferase, seems to have a regulatory role during osteoclast differentiation^27^. The SNP rs6722613 of this gene, not previously correlated with BMD, was associated with FN-sBMD, and in a logistic regression study, A/G-G/G genotype women showed increased risk of hip osteoporosis or osteopenia. Therefore, genetic variation in the *DNMT3A* gene must be taken into account when explaining total phenotypic variation in the BMD.

The *CARM1* gene codes for a histone-arginine methyltransferase functioning as both a coactivator and a methyltransferase^34^, which methylates both histones and non-histone substrates and which modulates gene expression by interacting with several transcription factors, and is overexpressed in different types of cancer^35–37^. Interestingly, *CARM1* expression has been described as higher in GG homozygous individuals (SNP rs12460421) than in other genotypes (^38^, and GTEx Portal, https://www.gtexportal.org), and as involved in carbohydrate-induced insulin secretion via the methylation of arginine 17 of histone H3 in pancreatic b cells^32^. If this is the case, it is reasonable to assume that women with GG genotype for the SNP of the *CARM1* gene could have experienced lifelong elevated plasma insulin levels after carbohydrate ingestion. Although the bone effects of hyperinsulinemia have not been completely elucidated^39^, several data support the hypothesis that insulin is anabolic in bone. Both osteoblasts and osteoclasts have insulin receptors^40,41^; in in vitro studies, physiological levels of insulin have been shown to increase OBs proliferation and function^42^, and in clinical studies insulin level has been reported as positively associated with BMD^39,43,44^. Consequently, lifelong increased insulin levels in GG genotype women could explain their higher BMD, consistent with the findings of the present work.

Although this same reasoning could be applied to explain the higher BMI observed in women homozygous for the G allele of the SNP of *CARM1* gene, there is a chicken-and-egg debate about whether obesity or insulin resistance has primacy; that is to say, whether the carbohydrate-insulin model of obesity is an established fact, or whether on the contrary fasting hyperinsulinemia could be driven by obesity-induced insulin resistance^45^. In this regard, recently published Mendelian randomization analysis provides strong evidence in favor of the carbohydrate-insulin model of obesity^46^, and the data we provide in this paper support the same hypothesis. The genetic variant (SNP of *CARM1* gene) is associated with higher insulin levels and higher BMI, which could indicate a causal relationship between increased insulin levels and BMI, analogous to the reasoning of the Mendelian randomization analysis.

Two of our SNPs deviated from HWE, despite a thorough review of genotypes. Several factors could explain this departure without resorting to a defective genotyping explanation^47,48^. When the entire cohort was analyzed (before applying the exclusion criteria and with a larger sample size of 1244 women), these *p* values were increased (rs9388451 of *HEY2* gene, *p* = 0.09; and rs12460421of *CARM1* gene, *p* = 0.012). However, given the strong association of the SNP of the *CARM1* gene with BMI, when only subjects with a normal BMI (18.5–24.9) were analyzed, both SNPs met HWE (*p* = 0.43 for *HEY2* SNP and *p* = 0.64 for *CARM1* SNP) indicating that the cause of HWE departure is enriched presence of the G allele of the *CARM1* gene in overweight and obese women.

Our study has some limitations. First, in cellular analysis study we compared the overall gene expression of OBs obtained from women with fragility fracture and women with osteoarthritis (as control case). Attaining the perfect control is precluded in this study by the difficulty of obtaining femur head bone samples in healthy women of similar age to the cases. Nevertheless, since osteoarthritis is primarily a disease of the joint rather than the bone, OBs obtained from femoral heads of osteoarthritic women can be considered reliable as control. Another drawback is that both the fragility fracture cohort and the BMD cohort are Caucasian women, so the data obtained in the present study may not be fully extrapolated to other populations or even to men. Finally, the sample size of the association study is medium, while that of the microarray study is low; we lack data on the diet and physical activity of the participating women; and although the present study is well powered, our findings have not been replicated in other cohorts. However, the GIANT study^49^ described a nominal association (*p* = 0.002) with BMI for G allele of the SNP rs12460421 of *CARM1* gene, which at least partly corroborates the association detected in this study.

In conclusion, in the present work we demonstrate that two SNPs in the *DNMT3A* and *CARM1* genes, both related to epigenetic processes, are associated with bone mass. Most significantly, the SNP rs12460421 of the *CARM1* gene was strongly associated with BMI and insulin levels, which can have important implications when assessing cardiovascular risk.

## Supplementary information

Supplementary Information 1.

Supplementary Information 2.

Supplementary Information 3.

Supplementary Information 4.

Supplementary Information 5.

## Data Availability

The raw microarray data is available in the Gene Expression Omnibus (GEO) repository (accession number GSE 156508). Restrictions apply to the availability of data generated or analyzed during this study to preserve patient confidentiality. However, upon reasoned request to the corresponding author and the Ethics Committee, this data may be transferred to interested researchers if the Ethics Committee, based on this reasoned request, gives its approval.
