# Supplementary figures and images for: Comparative transcriptome analysis identifies CARM1 and DNMT3A as genes associated with osteoporosis
Source: Sci Rep. 2020 Oct 1;10:16298. doi: 10.1038/s41598-020-72870-2 (PMC7530982; doi:10.1038/s41598-020-72870-2)

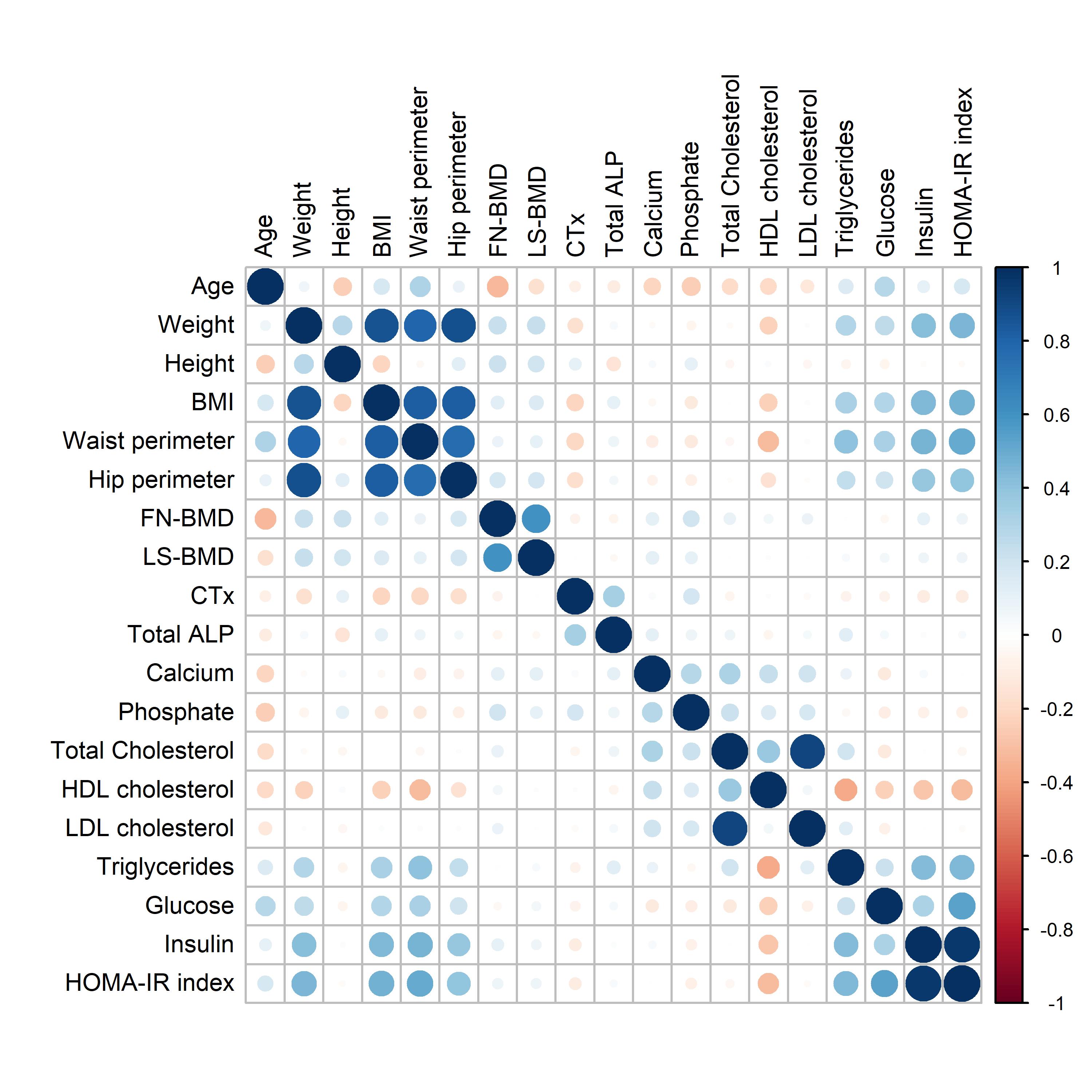

Supplement: Supplementary file 1 — Supplementary Information 1. [file 41598_2020_72870_MOESM1_ESM.jpg]
