# Supplementary material for: Comparative transcriptome analysis identifies CARM1 and DNMT3A as genes associated with osteoporosis
Source: Sci Rep. 2020 Oct 1;10:16298. doi: 10.1038/s41598-020-72870-2 (PMC7530982; doi:10.1038/s41598-020-72870-2)
Supplement: Supplementary file 2 — Supplementary Information 2. [file 41598_2020_72870_MOESM2_ESM.docx]

**Supporting Information**

**Comparative transcriptome analysis identifies *CARM1* and *DNMT3A* as genes**

**associated with osteoporosis**

Layla Panach ^1*^, Clara Pertusa ^1*^, Beatriz Martínez-Rojas ^1^, Álvaro Acebrón ^2^, Damián Mifsut ^2^, Juan J. Tarín ^3^, Antonio Cano ^4^, and Miguel Ángel García-Pérez ^1,5^

^1^ Research Unit, INCLIVA Health Research Institute, 46010-Valencia, Spain

^2^ Orthopedic Surgery and Traumatology, Clinic Hospital, INCLIVA Health Research Institute, 46010-Valencia, Spain

^3^ Department of Cellular Biology, Functional Biology and Physical Anthropology, University of Valencia, 46100-Burjassot, Spain

^4^ Department of Pediatrics, Obstetrics and Gynecology, University of Valencia, 46010-Valencia, Spain

^5^ Department of Genetics, University of Valencia, 46100-Burjassot, Spain

^*^ These authors contributed equally to this work

**Corresponding addres:**

**Dr. Miguel Ángel García-Pérez,** e-mail: migarpe@uv.es

**CONTENT**

**-Supplemental Table S1.** Sequence information of the primer sets (PCR, extension and competitors) for the MassARRAY gene expression analysis (Sequenom) of the 12 selected genes and 4 endogenous controls.

-**Supplemental Table S2**. Genes whose expression was significantly different between osteoblasts of Fracture and Control women (six women each).

-**Supplemental Table S3**. Gene Ontology-based biological process pathways altered after comparison of Fracture and Control RNA expression data.

-**Supplemental Table S4**. PANTHER analysis of differentially expressed genes.

**Supplemental Figure S1.** HeatMap displaying Pearson correlation coefficients between the variables measured in our cohort. The correlation matrix and its visual representation were obtained in R (Version 3.6.2, GNU project, The R Project for Statistical Computing).

**Supplemental Table S1.** Sequence information of the primer sets (PCR, extension and competitors) for the MassARRAY gene expression analysis (Sequenom) of the 12 selected genes and 4 endogenous controls.

| *MEF2C* LEFT | ACGTTGGATGCACCTACATAACATGCCACC |
| --- | --- |
| *MEF2C* RIGHT | ACGTTGGATGTTTGAGTAGAAGGCAGGGAG |
| *MEF2C* EXT | GTCAGTTGGGAGCTTGC |
| *THSD4* LEFT | ACGTTGGATGTGACATGGTTGGTCATGCAC |
| *THSD4* RIGHT | ACGTTGGATGTGCCAAGAGCTGGTTCCTCA |
| *THSD4* EXT | CCGCTGAGCACCTTTCGC |
| *SULF2* LEFT | ACGTTGGATGTTATGCAGCTTCAGCTTCCC |
| *SULF2* RIGHT | ACGTTGGATGTGTGAAGGACCTGTGTCAGC |
| *SULF2* EXT | TCACTGCCACTTCTGTCCC |
| *ITGA4* LEFT | ACGTTGGATGTTGATCACTGAAGCGTTGGC |
| *ITGA4* RIGHT | ACGTTGGATGAACACGCTGTTCGGCTACTC |
| *ITGA4* EXT | ACCCACTAGGAGCCATCGG |
| *DSG3* LEFT | ACGTTGGATGGTTGTTCATAATCTAGAGCC |
| *DSG3* RIGHT | ACGTTGGATGCTGGGAATGAAGGAAATTGG |
| *DSG3* EXT | CATAATCTAGAGCCTTCACC |
| *RSPO3* LEFT | ACGTTGGATGTTTCTTTTCCTCTCCCTTCC |
| *RSPO3* RIGHT | ACGTTGGATGTGTGTCCCCCAACAAATGAG |
| *RSPO3* EXT | ACTTCCTTTTTTTCCTCGTTC |
| *IL13RA2* LEFT | ACGTTGGATGTGCGTAAGCCAAACACCTAC |
| *IL13RA2* RIGHT | ACGTTGGATGGCCATGACTGGAAACTGTTG |
| *IL13RA2* EXT | ACACCTACCCAAAAATGATTCC |
| *COL10A1* LEFT | ACGTTGGATGAGCTTCAGAAAGCTGCCAAG |
| *COL10A1* RIGHT | ACGTTGGATGGATACTAGCAGCAAAAAGGG |
| *COL10A1* EXT | CGAACTCCCAGCACGCAGAATCCA |
| *LEP* LEFT | ACGTTGGATGGTAGGAATCGCAGCGCCAG |
| *LEP* RIGHT | ACGTTGGATGAAAGCCACAAGAATCCGCAC |
| *LEP* EXT | AGCCAGCGGTTGCAAGGCCCAAGA |
| *ADAM12* LEFT | ACGTTGGATGAACTTGTCACAGAAGGGAGG |
| *ADAM12* RIGHT | ACGTTGGATGTTCACGAGTGTGCAATGCAG |
| *ADAM12* EXT | TTCTTCCTGTTGTTGCACACCCCTC |
| *GALNT5* LEFT | ACGTTGGATGCTTCCAGAATGACTGTAGTG |
| *GALNT5* RIGHT | ACGTTGGATGTGACTTAAGGGCTCCCATTG |
| *GALNT5* EXT | CCCAAAGCCACATTAATAAGCACACC |
| *FOSL1* LEFT | ACGTTGGATGAACCGGAGGAAGGAACTGAC |
| *FOSL1* RIGHT | ACGTTGGATGTTCTGCAGCTCCTCAATCTC |
| *FOSL1* EXT | GTGACCGACTTCCTGCAGGCGGAGAC |
| **Endogenous genes** | |
| *GAPDH* LEFT | ACGTTGGATGATGGTGTCTGAGCGATGTGG |
| *GAPDH* RIGHT | ACGTTGGATGTATAAATTGAGCCCGCAGCC |
| *GAPDH* EXT | CGATGTGGCTCGGCTGGCG |
| *HPTR1* LEFT | ACGTTGGATGCCCATCTCCTTCATCACATC |
| *HPTR1* RIGHT | ACGTTGGATGTGCTGAGGATTTGGAAAGGG |
| *HPTR1*-4 EXT | AAAGACGTTCAGTCCTGTCCA |
| *ACTB* LEFT | ACGTTGGATGAAGGCCAACCGCGAGAAGAT |
| *ACTB* RIGHT | ACGTTGGATGGGATAGCACAGCCTGGATAG |
| *ACTB* EXT | GAGAAGATGACCCAGATCATG |
| *HMB2* LEFT | ACGTTGGATGATGTTACGAGCAGTGATGCC |
| *HMB2* RIGHT | ACGTTGGATGCATACAAGAGACCATGCAGG |
| *HMB2* EXT | TCAGGGCCATCTTCATGCTGGGC |
| **COMPETITOR PRIMERS** | |
| *MEF2C* COMP | TTTGAGTAGAAGGCAGGGAGAGATTTGAACTCTGAGATAAATGAGTGCTAGaGCAAGCTCCCAACTGACTGAGGGCAGATGGTGGCATGTTATGTAGGTG |
| *THSD4* COMP | TGACATGGTTGGTCATGCACACCACCGAGCGTGTCCGCACTCCGGCCCCACACTCCGCTGAGCACCTTTCGCaCCACTCGGTGAGGAACCAGCTCTTGGCA |
| *SULF2* COMP | TTATGCAGCTTCAGCTTCCCCGTGGCGTCCTCCACACACTGCCACTTCTGTCCCtGCTGCTCACACGCCGTCTGGTACTCAGCACGCTGACACAGGTCCTTCACA |
| *ITGA4* COMP | TTGATCACTGAAGCGTTGGCGAGCCAGTTGGCAGTGGGCGCACCCACTAGGAGCCATCGGaTCGCCCCGTGGCTGTGCAGCACGACCGAGTAGCCGAACAGCGTGTT |
| *DSG3* COMP | GTTGTTCATAATCTAGAGCCTTCACCTCTTTCAGGATGCCTTCATTAGTTCTAGGATCAGTTTGTATTTCAAACCAATTTCCTTCATTCCCAG |
| *RSPO3* COMP | TTTCTTTTCCTCTCCCTTCCTTTTTTTCCTCGTTCaCCCTTCTGACACTTCTTCCTTTGCACTGTACACTTTCTTGTCTCATTTGTTGGGGGACACA |
| *IL13RA2* COMP | GCCATGACTGGAAACTGTTGAGTCAATACCATGTCTCTTGATATGGAAAGTCTTCATGTATCACAGAAAAATTCaGGAATCATTTTTGGGTAGGTGTTTGGCTTACGCA |
| *COL10A1* COMP | GATACTAGCAGCAAAAAGGGTATTTGTGGCAGCATATTCTCAGtTGGATTCTGCGTGCTGGGAGTTCCTGGAGATGGTGCCTTGGCAGCTTTCTGAAGCT |
| *LEP* COMP | AAAGCCACAAGAATCCGCACAGGGTTCCCCAATGCATTTTCCTTCCCAGGATGGGCaTCTTGGGCCTTGCAACCGCTGGCGCTGCGATTCCTAC |
| *ADAM12* COMP | AACTTGTCACAGAAGGGAGGTGCCCAGTGGGCCTCGCAGTGGCAGTTCTTCCTGTTGTTGCACACCCCTCaGCCGTGGCACTGCATTGCACACTCGTGAA |
| *GALNT5* COMP | CTTCCAGAATGACTGTAGTGTTTTCAATGGAAATGCATTTACCCAAAGCCACATTAATAAGCACACCtCTAGCTCTCACAATGGGAGCCCTTAAGTCA |
| *FOSL1* COMP | TTCTGCAGCTCCTCAATCTCTCGCTGCAGCCCAGATTTCTCATCTTCCAGTTTGTCtGTCTCCGCCTGCAGGAAGTCGGTCAGTTCCTTCCTCCGGTT |
| **Endogenous genes** | |
| *GAPDH* COMP | ATGGTGTCTGAGCGATGTGGCTCGGCTGGCGtCGCAAAAGAAGATGCGGCTGACTGTCGAACAGGAGGAGCAGAGAGCGAAGCGGGAGGCTGCGGGCTCAATTTATA |
| *HPTR1* COMP | CCCATCTCCTTCATCACATCTCGAGCAAGACGTTCAGTCCTGTCCAaAATTAGTCCATGAGGAATAAACACCCTTTCCAAATCCTCAGCA |
| *ACTB* COMP | GGATAGCACAGCCTGGATAGCAACGTACATGGCTGGGGTGTTGAAGGTCTCAAtCATGATCTGGGTCATCTTCTCGCGGTTGGCCTT |
| *HMB2* COMP | ATGTTACGAGCAGTGATGCCTACCAACTGTGGGTCATCCTCAGGGCCATCTTCATGCTGGGCtGGGACATGGATGGTAGCCTGCATGGTCTCTTGTATG |
